# NMR resonance assignments of the four isoforms of the hazelnut allergen Cor a 1.04

**DOI:** 10.1007/s12104-019-09918-6

**Published:** 2019-11-05

**Authors:** Sebastian Führer, Ricarda Zeindl, Martin Tollinger

**Affiliations:** Institute of Organic Chemistry, Center for Molecular Biosciences Innsbruck (CMBI), University of Innsbruck, Innrain 80/82, 6020 Innsbruck, Austria

**Keywords:** NMR resonance assignment, TALOS + prediction, PR-10 protein, Cross-reactivity, Allergen

## Abstract

In large parts of Europe, Northern America and China people are suffering from allergies after consuming certain kinds of fruits and vegetables. Typical allergic symptoms range from scratching and itching of the throat to severe symptoms like rhino conjunctivitis and anaphylaxis. For hazelnuts (*Corylus avellana*), these allergies result from initial sensitization to the birch (*Betula verrucosa*) pollen allergen Bet v 1 and subsequent development of allergic cross-reactions to proteins that are similar in their three-dimensional structure to the sensitizing protein Bet v 1. The cross-reactive proteins in hazelnut are the four isoforms Cor a 1.04 with a molecular weight of about 17.5 kDa. Significant differences regarding the immunologic behavior of these proteins have been reported. In this work we assigned backbone and side chain ^1^H, ^13^C, and ^15^N chemical shifts of these four isoforms, Cor a 1.0401, Cor a 1.0402, Cor a 1.0403, and Cor a 1.0404 by solution NMR spectroscopy. The chemical shift data confirm the characteristic Bet v onefold for all four isoforms, consisting of seven β-strands that are separated by two short α-helices, along with a long C-terminal α-helix. These data provide the basis for a comparative structural and dynamic analysis of these proteins by NMR in order to characterize their different immunologic cross-reactivities on a molecular level.

## Biological context

In the northern hemisphere, pollinosis caused by sensitization to birch pollen is the most common allergy. In 62–98% of birch pollen allergic patients IgE-specific antibodies are directed at the protein Bet v 1, the major birch pollen allergen (Ipsen and Lowenstein 1983; Moverare et al. 2002). Immunological cross-reactivity of Bet v 1 specific antibodies (IgE) to proteins that are present in fruits and nuts can provoke additional allergic reactions in patients suffering from birch pollinosis. These foods typically trigger oral allergic syndromes (OAS), including itching and scratching of the oral cavity, directly after consumption (Mari et al. 2005). Allergic reactions to raw hazelnuts are among the most prevalent manifestations of these birch pollen related food allergies, affecting 60–70% of all individuals, who are sensitized towards birch pollen (Geroldinger-Simic et al. 2011; Hansen et al. 2009).

Allergic cross-reactivities are associated with the class 10 of pathogenesis related (PR) proteins in hazelnut and hazel pollen (Vieths et al. 2002), whose expression is induced by environmental or pathogenic stress. These allergens consist of about 160 amino acid residues with a molecular weight of ~ 17.5 kDa. The canonical fold of PR-10 proteins comprises seven antiparallel β-strands (β1–β7), which are interrupted by two short α-helices (α1 and α2) between strands β1 and β2. In addition, the β-sheet is covered by a long C-terminal α-helix (Fernandes et al. 2013). It was shown that allergic cross-reactions decrease after roasting of the hazelnuts, which indicates the general heat lability of these proteins (Verhoeckx et al. 2015). The PR-10 proteins found in hazel pollen are grouped into the Cor a 1.01 isoforms (Breiteneder et al. 1993), while Cor a 1.04 isoforms are found in the hazelnut. Four Cor a 1.04 isoforms have been identified (Hirschwehr et al. 1992) and their IgE-binding capacity was studied in detail (Lüttkopf et al. 2002). These isoforms are Cor a 1.0401 (AF136945), Cor a 1.0402 (AF323973), Cor a 1.0403 (AF323974), and Cor a 1.0404 (AF323975) (Hirschwehr et al. 1992), which share a sequence identity of about 97% among each other, and only about 63 and 66% to the pollen allergens Cor a 1.01 and Bet v 1.0101 (Gajhede et al. 1996), respectively. The PR-10 food allergens from apple (*Malus* domestica) Mal d 1.0101 (Ahammer et al. 2017, 2016) and peach (*Prunus 0persica*) Pru p 1.0101 (Führer et al. 2019), which show the highest birch pollen associated food hypersensitivity along with Cor a 1.04, have sequence identities of 62–64% to the four Cor a 1.04 isoforms. Using an enzyme allergosorbent test, it was shown that the various Cor a 1.04 proteins significantly differ with respect to their IgE-reactivity, resulting in the immunological ranking Cor a 1.0401 > 02 > 03 > 04 (Lüttkopf et al. 2002). In this work we present the solution NMR backbone and side-chain assignments of the four recombinantly expressed isoforms of Cor a 1.04.

## Methods and experiments

### Sample preparation

Transformation of the codon-optimized plasmids of Cor a 1.0401 (GenBank nucleotide code AF136945 and protein code AAD48405), Cor a 1.0402 (GenBank nucleotide code AF323973 and protein code AAG40329), Cor a 1.0403 (GenBank nucleotide code AF323974 and protein code AAG40330), and Cor a 1.0404 (GenBank nucleotide code AF323975 and protein code AAG40331), which were cloned in the expression vector pET28b (+) using restriction enzymes NcoI and XhoI, was conducted in the *E. coli* strain BL21(DE3) Star (Invitrogen). An overnight culture (100 mL) of Luria–Bertani (LB) medium with 25 µg/mL kanamycin was inoculated with one bacterial colony and incubated overnight at 37 °C and 200 rpm. The volume of the overnight culture, which was centrifuged at 2000×*g*, to reach a cell density of 0.1 in the final expression culture, was calculated by V_o/n_ = (0.1 × V_expression_)/A_600, o/n_. The cell pellet was resuspended in 1 L of M9 minimal medium enriched with 1 g/L ^15^NH_4_Cl or 3 g/L ^13^C_6_-d-glucose and 1 g/L ^15^NH_4_Cl (both Cambridge Isotope Laboratories) and supplemented with 25 µg/mL kanamycin. Additionally, 1 g/L ISOGRO^®^—^15^N or 1 g/L ISOGRO^®^—^13^C, ^15^N powder (both Sigma-Aldrich) was added to the medium. The culture was incubated at 37 °C and 200 rpm until the cell density reached 0.5–0.6 (at 600 nm), subsequently protein expression was induced by addition of isopropyl-β-D-1-thiogalactopyranosid (IPTG, 1 mM) and performed for 3 h at 37 °C. Cells were harvested at 3440×*g* and 4 °C for 35 min, resuspended in a buffer containing 25 mM imidazole, 0.1% Triton X-100, and 0.5 M urea, shock-frozen in liquid nitrogen and stored at − 80 °C until usage. Lysate preparation was done by thawing the cells and pre-treating for 1 h on ice with lysozyme (10 µg/mL) and only for Cor a 1.0404 additionally with 300 µl protease inhibitor cocktail His-tag (Carl Roth) per 50 mL suspension. Afterwards, DNAse (1 µg/mL) was added and the cells were passed through a French Press and centrifuged at 15,000×*g* and 4 °C for 35 min. The cleared lysate was loaded onto an anion exchange column (Resource Q 6 mL, GE Healthcare) and the desired Cor a 1.04 proteins were eluted with a sodium chloride gradient over 30 mL from 0 to 50% in 25 mM TrisHCl buffer (pH 7.5) at a flow rate of 2 mL/min. Cor a 1.04 containing fractions were collected and concentrated to about 1.5 mL by centrifugation (Amicon Ultra 3 kDa MWCO, Merck Millipore). For the final purification step the corresponding protein was loaded onto a size exclusion column (HiLoad 16/600 Superdex 75 prep grade, GE Healthcare) and eluted isocratically at 1 mL/min with a 10 mM sodium phosphate buffer (pH 6.9) containing 2 mM DTT. All purification steps were monitored by SDS-PAGE gel electrophoresis with 15% gels. Samples were supplemented with 10% D_2_O (v/v) for NMR spectroscopy, yielding concentrations of 0.5 mM for ^15^N labeled and ^15^N/^13^C labeled Cor a 1.04 proteins.

### NMR spectroscopy

A 500 MHz Agilent DirectDrive 2 spectrometer equipped with a room temperature probe was used to record all NMR spectra at 25 °C. Backbone resonance assignments were performed using a two-dimensional ^1^H-^15^N-HSQC and three-dimensional HNCACB, CBCA(CO)NH, HNCO, and HN(CA)CO experiments. A two-dimensional ^1^H-^13^C-HSQC and three-dimensional (H)CC(CO)NH-TOCSY, H(CCO)NH-TOCSY, ^1^H-^15^N-TOCSY-HSQC, ^1^H-^15^N-NOESY-HSQC, and ^1^H-^13^C-NOESY-HSQC experiments were used to perform side-chain assignments. Assignment of the aromatic side-chains of phenylalanines, tyrosines and histidines was obtained from aromatic ^1^H-^13^C-HSQC experiments, a three-dimensional aromatic ^1^H-^13^C-NOESY-HSQC experiment and a ^1^H-^15^N-HSQC experiment with coherence transfer optimized for ^2^*J* couplings in imidazole side-chains of histidines. Data processing was performed with NMRPipe (Delaglio et al. 1995) and the CcpNMR software package was used for resonance assignment (Vranken et al. 2005).

### Assignments and data deposition

**Fig. 1:**
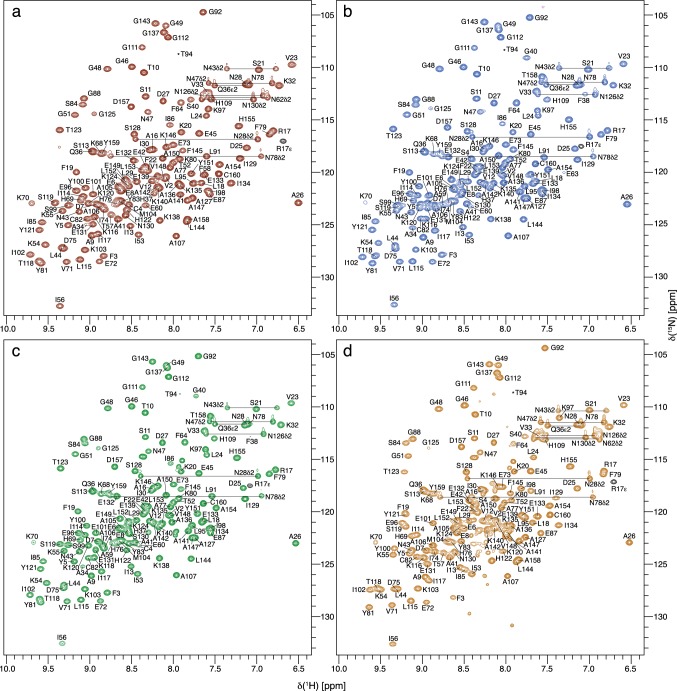
500 MHz ^1^H-^15^N-HSQC spectrum of **a** Cor a 1.0401, **b** Cor a 1.0402, **c** Cor a 1.0403, and **d** Cor a 1.0404 (each 0.5 mM) in 20 mM sodium phosphate (pH 6.9) and 2 mM DTT, supplemented with 10% D_2_O at 25 °C. Assigned residues are indicated by single letter codes and *horizontal lines* indicate asparagine and glutamine NH_2_ side-chain resonances. The signals labeled by an *asterisk* indicates the positions of residues below the intensity cut-off. Resonance assignments are available online at the BMRB repository (Accession numbers for Cor a 1.0401, Cor a 1.0402, Cor a 1.0403, and Cor a 1.0404 are 27,965, 27,961, 27,967, and 28,016, respectively)

We were able to assign 138 of 152 non-proline residues for Cor a 1.0401 (Fig. 1a), 139 of 152 non-proline residues for Cor a 1.0402 (Fig. 1b), 138 of 152 non-proline residues for Cor a 1.0403 (Fig. 1c), and 132 of 151 non-proline residues for Cor a 1.0404 (Fig. 1d). The ^1^H-^15^N-HSQC spectra of Cor a 1.0401–03 show well folded proteins with very similar shift distributions, in agreement with their high sequence identities. The spectrum of the least allergenic isoform Cor a 1.0404 shows additional peaks in ^1^H-^15^N-HSQC spectra, which probably arise from partial protein unfolding or degradation despite the use of protease inhibitors (Table 1).

**Table 1:** Completeness of backbone and side-chain resonance assignments for the four different isoforms of Cor a 1.04

	Cor a 1.0401 (%)	Cor a 1.0402 (%)	Cor a 1.0403 (%)	Cor a 1.0404 (%)
C’	94.4	95.0	94.4	90.0
C^α^	95.0	95.6	94.4	90.6
C^β^	97.2	95.8	95.1	92.4
C^γ^	72.6	70.1	71.6	68.4
C^δ^	78.8	75.8	76.8	72.0
C^ε^	71.4	69.0	76.2	61.9
H	91.4	92.1	91.4	88.0
H^α^	92.6	92.1	91.5	86.9
H^β^	93.1	91.8	92.2	87.8
H^γ^	88.8	85.7	90.1	79.0
H^δ^	93.4	88.1	92.4	85.5
H^ε^	69.4	72.6	74.2	64.5
N	90.8	91.4	90.8	87.4
N^δ^	85.7	83.3	83.3	85.7
N^ε^	77.8	77.8	77.8	77.8

Assignment for the backbone amide corresponds to non-proline residues

**Fig. 2:**
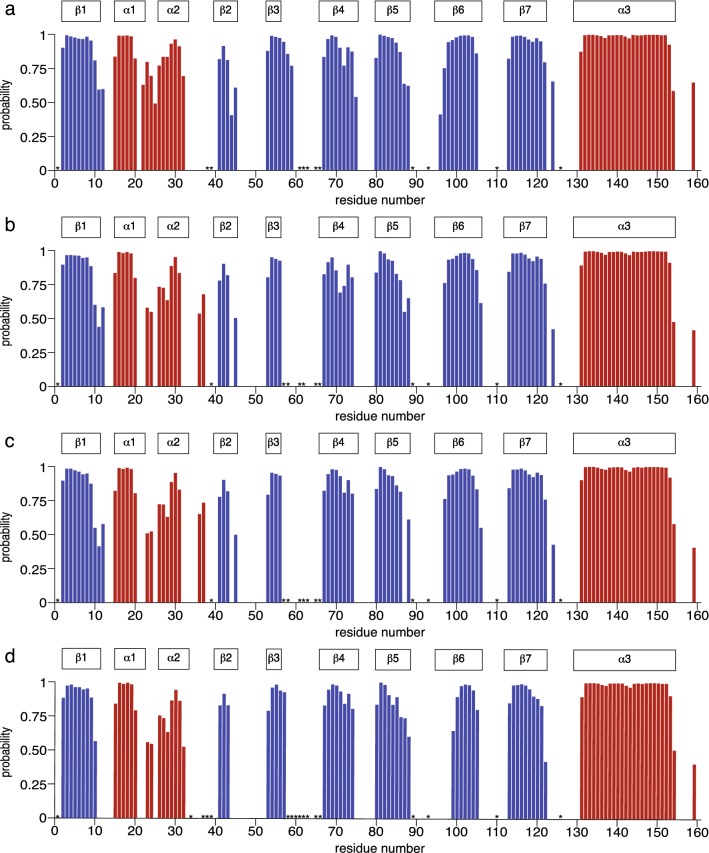
Secondary structure of **a** Cor a 1.0401, **b** Cor a 1.0402, **c** Cor a 1.0403, and **d** Cor a 1.0404 as predicted by TALOS + , based on backbone H^N^, N, C’, C^α^, and C^β^ chemical shifts. Secondary structure probabilities (*red*, α-helices; *blue*, β-strands) are plotted *versus* residue numbers. *Asterisks* indicate residues for which backbone amide NH resonance assignments are not available. Secondary structure elements of Bet v 1.0101 (PDB: 4A88) are indicated on *top*

The accession numbers at the Biological Magnetic Resonance Data Bank (http://www.bmrb.wisc.edu) for Cor a 1.0401, Cor a 1.0402, Cor a 1.0403, and Cor a 1.0404 are 27,965, 27,961, 27,967, and 28,016, respectively. Based on the H^N^, N, C’, C^α^, and C^β^ backbone chemical shifts, the TALOS + software (Shen et al. 2009) was used to predict the secondary structure elements of the four Cor a 1.04 isoforms (Fig. 2). These data are in agreement with the PR-10 fold, containing seven Q-strands (β1–β7) that are interrupted by two short α-helices (α1 and α2) and a long C-terminal α-helix. An additional propensity for α-helical structure is found for the segment between α2 and β2 in two isoforms, while a moderate α-helical propensity is also present after the C-terminal helix in all four proteins. In some loop regions, particularly between α2/β2, β3/β4, and β5/β6, several resonances are absent in ^1^H-^15^N-HSQC and triple-resonance spectra of all four isoforms, possibly due to conformational exchange or due to exchange of backbone amides with solvent.

The NMR resonance assignment of Cor a 1.0401, Cor a 1.0402, Cor a 1.0403, and Cor a 1.0404 obtained in this work will enable us to analyze structural and dynamic properties of these proteins in detail in a comparative manner, and to relate these properties to the previously observed differences in their immunological reactivities.
